# speed-induced passive depth stabilization in a biomimetic underwater vehicle with a swim bladder

**DOI:** 10.1038/s41598-026-56420-w

**Published:** 2026-08-04

**Authors:** Nazar Ismailov, Nikolay Tschur, Georgii Kazantsev, Sergey Lobov, Victor Kazantsev

**Affiliations:** 1 Moscow Institute of Physics and Technology (National Research University), Dolgoprudny, Russia; 2 Russian State Scientific Center for Robotics and Technical Cybernetics, Saint Petersburg, Russia; 3 NEIMARK University, Nizhny Novgorod, Russia

**Keywords:** Engineering, Physics

## Abstract

A nonlinear mathematical model describing the vertical motion of a biomimetic underwater vehicle equipped with a swim bladder is developed. For a passive bladder that changes its volume under hydrostatic pressure, the system can achieve depth stabilization through a speed-induced mechanism when the lever-arm geometry (relative positions of the swim bladder and lifting surfaces) is favorable; otherwise stabilization is not possible. Using the Routh–Hurwitz criterion, analytical stability conditions are obtained in closed form, revealing a lower onset speed that is set by a simple coupling between forward speed and geometry. Numerical simulations confirm the theoretical predictions and reveal the dominant loss-of-stability scenarios: loss of effective stiffness at the onset threshold and oscillatory instability when the mixed speed–geometry factor changes sign. The results demonstrate the feasibility of passive swim-bladder-based depth stabilization and provide practical guidelines for selecting the lever arms of the buoyancy and lift forces and operating speeds in autonomous underwater vehicles.

## Introduction

Precise depth control and stabilization represent a critically important task for a wide range of underwater systems–from biological organisms to autonomous underwater vehicles (AUVs)^1–5^.

In nature, teleost fishes solve this problem using a swim bladder, which combines passive and active mechanisms for buoyancy regulation^6^. As early as the 19th century, Moreau^7^ demonstrated that, counterintuitively, fish with a swim bladder are in a state of unstable equilibrium. This instability arises from the passive change in gas volume within the bladder with depth, according to Boyle’s law. The swim bladders of most fish species are well-described by Boyle’s law, although in some, such as cyprinids (Cyprinidae), bladder volume is less sensitive to depth due to high internal pressure and wall stiffness^8^. On the other hand, the active physiological mechanism of gas exchange between the blood and the swim bladder, primarily facilitated by the gas gland and the rete mirabile vascular structure, allows for the regulation of gas volume to maintain neutral buoyancy at different depths^6^. However, these processes are slow, taking hours, with the result that species undertaking significant vertical migrations may remain in a state far from neutral buoyancy for extended periods^9,10^.

Thus, the swim bladder alone does not ensure a stable position of the fish in the water column and requires additional dynamic stabilization mechanisms–both active and passive. An example of an active mechanism is the oscillation of pectoral fins, generating compensatory movements^3^. If the fish is in forward motion, the lift force acting on the body’s lifting surfaces and fins can provide a passive stabilization mechanism^1^. This mechanism is particularly pronounced in high-speed species such as tunas^2^.

Another problem associated with the presence of a swim bladder is the emergence of a pitching moment and, consequently, pitch instability when the center of mass and the center of buoyancy do not coincide^1,11,12^. In this case as well, dynamic stabilization–active or passive–plays a key role.

In robotics, bio-inspired propulsion and control methods have been extensively developed to mimic the efficiency and maneuverability of aquatic animals. For rhythmic locomotion in robotic fish, Central Pattern Generators (CPG) have become a standard approach for generating adaptive motion patterns^13–16^. A promising concept of self-organizing CPGs emerging from the repetition of circulating neural activity and STDP synaptic plasticity has been explored in^17,18^. Comprehensive research on modeling and control of biomimetic underwater vehicles, such as the work by Plamondon^19^, highlights the complexities of active control in such systems and establishes a benchmark for dynamic modeling and controller synthesis. Furthermore, modern surveys, such as Wang et al.^14^, provide a systematic overview of actuation, design, and advanced control techniques for biomimetic underwater robots, covering both open-loop and closed-loop strategies for depth, orientation, trajectory tracking, and swarm coordination^20,21^. Recent advances also focus on resource-aware control strategies that optimize the trade-off between performance and communication load in networked systems^22^.

In contrast to the prevalent active control paradigms, which often require continuous energy expenditure for actuation and sensing, this study investigates a fundamentally passive mechanism for depth and pitch stabilization. While active methods, such as adaptive nonlinear controllers^23^ or event-triggered strategies^22^, excel in handling disturbances and varying conditions, they add complexity and resource demands. Our work aims to provide a foundational passive layer that could either operate independently in steady cruise conditions or be complemented by lightweight active controllers for maneuverability and disturbance rejection, forming a potential hybrid architecture.

The development of technical systems utilizing bio-inspired propulsion mechanisms appears to be a promising direction for creating energy-efficient and low-noise underwater robots^24,25^. However, in the field of depth control, the vast majority of existing solutions are based on ballast tank systems that pump ambient water [see, for example^26–28^]. Besides the need for relatively noisy pumps, such systems, strictly speaking, are not analogous to the swim bladder, as they regulate buoyancy by changing mass rather than volume. Only recently have studies emerged where the swim bladder principle is utilized in technical systems^29^ and robotic fish^30^. Concerning dynamic depth stabilization systems in AUVs, they are implemented in both active^31^ and passive^5,32^ forms.

In light of the outlined problems related to bio-imitation of buoyancy systems, this research focuses on a fundamental aspect–the development and analysis of a model for passive depth and pitch stabilization for an underwater vehicle equipped with an analogue of a swim bladder. We aim to test the hypothesis that forward motion can provide depth stability without continuous active control, similar to the case in some fish species. For this purpose, we derive analytical stability conditions linking the vehicle’s speed to its geometry and present them as a set of practically applicable design criteria. Our work provides a theoretical foundation and specific engineering guidelines for creating more energy-efficient and low-noise biomimetic robots.

## Mathematical model of the system

We consider planar (vertical-plane) motion of a streamlined body (fish or underwater vehicle) with mass *m* and pitch inertia *J*. The vertical axis *z* is directed upward; positive pitch *θ >0* corresponds to “nose up”. The vehicle advances at a constant forward speed *U>0*. Small angles are assumed throughout.

**Fig. 1:**
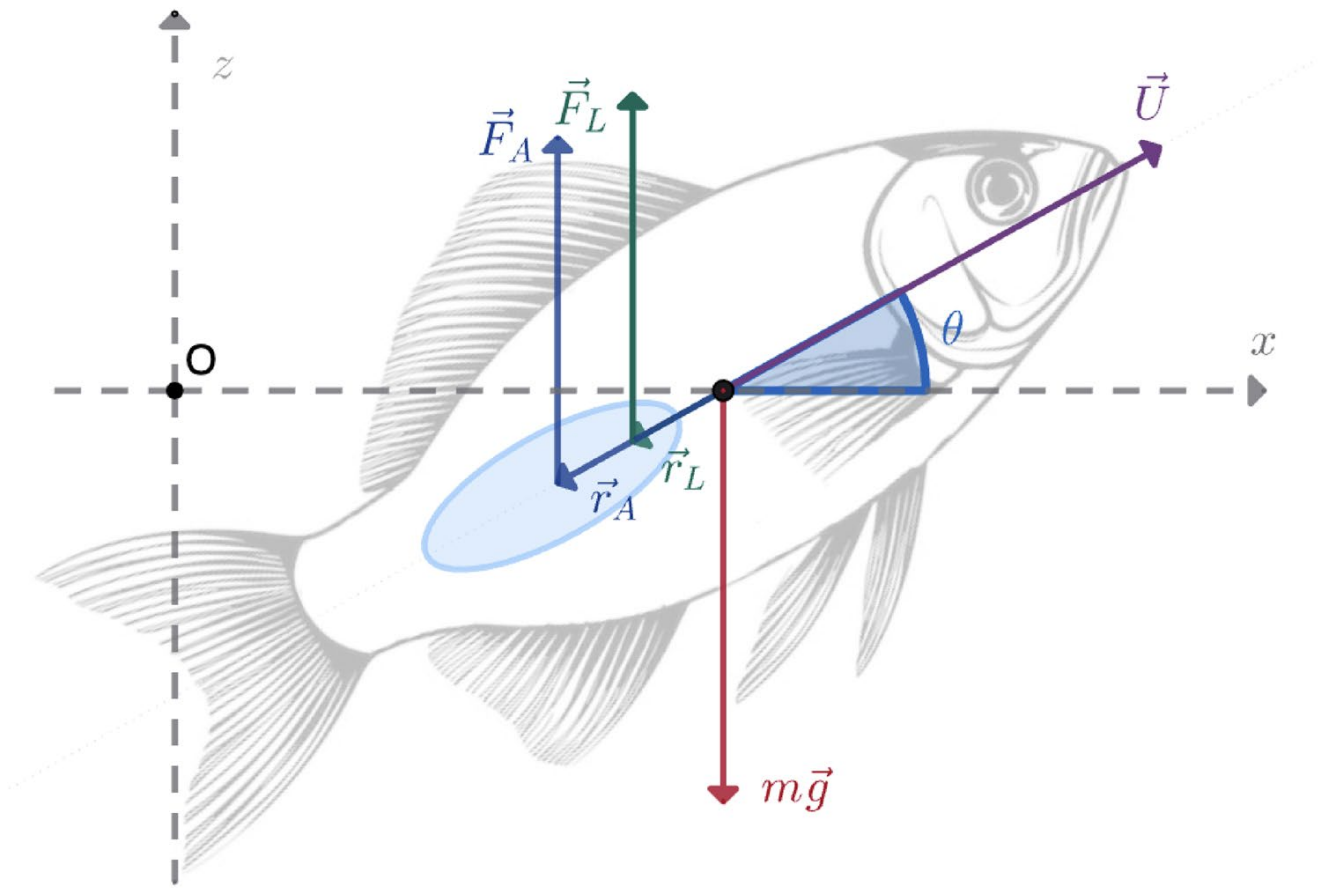
Schematic of the fish-with-tail-bladder model used in this paper. The bladder is placed at offset $r_A$ from the CM; the lift force $F_L$ acts at offset $r_L$.

A compliant swim bladder is positioned such that its center of buoyancy, which is the point of application of the net Archimedean force, is located at a longitudinal offset $r_A$ from the center of mass (CM), coaxial with the body axis (a tail-mounted bladder implies $r_A<0$). The resultant aerodynamic/hydrodynamic lift force, arising from both the body’s pitch and the action of lateral fins aligned with the body, acts at a longitudinal offset $r_L$ from the CM, also coaxial with the body axis; its sign is not restricted a priori (in particular, $r_L<0$ for a tail application point, $r_L>0$ for a forward one). Figure 1 illustrates the geometry.

At the operating depth the static balance is $mg=\rho _w g V_0$, where $\rho _w$ is water density and $V_0$ is the nominal bladder volume. Small variations of depth and pitch change the hydrostatic pressure at the bladder center located at height $z_b \approx z + r_A \theta$; with a polytropic gas law $pV^\kappa =\textrm{const}$ and hydrostatic pressure $p(z)=p_0+\rho _w g z$, the linearized buoyancy variation about equilibrium reads

1 $$\begin{aligned} \begin{aligned}&\delta F_A = k_b(z + r_A\theta ), \\&\delta M_A = r_A\delta F_A = r_A k_b z + r_A^2 k_b\theta , \end{aligned} \end{aligned}$$

with the (positive) “buoyancy stiffness”

2 $$\begin{aligned} k_b \equiv \frac{\rho _w^2 g^2 V_0}{\kappa p_0} > 0, \end{aligned}$$

computed at the operating point ($p_0$ is the reference pressure). This model is *passive*: the amount of gas in the bladder is fixed and there is no mass inflow or outflow.

For small angles the angle of attack satisfies $\alpha \simeq \theta - \dot{z}/U$. The vertical lift and its moment are modeled as

3 $$\begin{aligned} \begin{aligned}&F_L = K U^2\alpha = K U^2\theta - K U\dot{z},\\&M_L = r_LF_L = r_L K U^2\theta - r_L K U\dot{z}, \end{aligned} \end{aligned}$$

where *K>0* is an effective (aero)hydrodynamic lift coefficient that serves as a lumped parameter encompassing all design-specific characteristics of the body-fins system, including geometry, planform area, and other relevant factors. A linear structural pitch stiffness $-k_\theta \theta$ ($k_\theta >0$) is also included. To focus on the core stabilization mechanism, external viscous drag in the *z*-equation is not added; it does not alter the existence of the stability window established below.

Collecting forces and moments in the vertical plane yields the coupled second-order system:

4 $$\begin{aligned} \left\{ \begin{aligned} m\ddot{z}&= -KU\dot{z} + k_b z + (k_b r_A + K U^2)\theta ,\\ J\ddot{\theta }&= -r_L K U\dot{z} + r_A k_b z - (k_\theta - r_A^2 k_b - r_L K U^2)\theta . \end{aligned} \right. \end{aligned}$$

The physical interpretation of system (4) reveals the stabilization mechanism. In the vertical dynamics (*z*-equation), the term $-KU\dot{z}$ represents lift damping due to vertical motion (proportional to vertical velocity); $k_b z$ is the restoring buoyancy force from swim bladder compression/expansion; and $(k_b r_A + K U^2)\theta$ captures the combined effect of buoyancy-induced force due to pitch (via the offset $r_A$) and lift force due to pitch angle.

In the angular dynamics (*θ*-equation), the term $-r_L K U\dot{z}$ is the moment generated by the lift force during vertical motion; $r_A k_b z$ is the moment from the buoyancy force due to vertical displacement; and $-(k_\theta - r_A^2 k_b - r_L K U^2)\theta$ represents the net pitch stiffness combining structural stiffness ($k_\theta$), buoyancy-induced pitch stiffness ($-r_A^2 k_b$), and lift-induced pitch stiffness ($-r_L K U^2$).

The system dynamics are formulated in state-space representation by defining the state vector $x=(z,\dot{z},\theta ,\dot{\theta })^\top,$ which allows the governing equations to be expressed as the linear system $\dot{x}=A(U)x$, where *A*(*U*) is the state matrix that depends on the forward speed *U*.

The stability analysis of the system described by Eq. (4) was supported by numerical simulation to illustrate the resulting dynamic regimes. The simulations were performed using the parameter set listed in Table 1, which models a small-scale underwater vehicle. These physically plausible values were chosen to clearly demonstrate the key phenomena of depth stabilization and the emergence of dynamic instability, thereby providing a concrete numerical validation of the analytical framework.

**Table 1:** Model parameters for the linearized vehicle dynamics.

Parameter	Symbol	Value
Mass	*m*	1.0 kg
Pitch moment of inertia	*J*	1 kg*·*m^2^
Water density	$\rho _w$	1000 kg/m^3^
Reference pressure	$p_0$	$10^5$ Pa
Buoyancy stiffness	$k_b$	0.5 N/m
Lift coefficient	*K*	1 kg/m
Structural pitch stiffness	$k_\theta$	5 N*·*m/rad
Bladder offset from CM	$r_A$	− 3 m
Lift application offset	$r_L$	− 2 m

## Stability analysis

Linearizing the passive small-angle model about the equilibrium $(z,\dot{z},\theta ,\dot{\theta }) = (z^*,0,0,0)$ for a given forward speed *U > 0* yields the system $\dot{\boldsymbol{x}} = A(U)\boldsymbol{x}$, where $\boldsymbol{x} = (z,\dot{z},\theta ,\dot{\theta })^\top$. The Routh–Hurwitz criterion reveals two key speed thresholds governing stability:

5 $$\begin{aligned} \begin{aligned} U&> U_1, \quad U_1^2 = \frac{k_\theta }{K(r_L - r_A)} \quad (r_L> r_A), \\ U&> U_2, \quad U_2^2 = \frac{k_b(J + m r_L r_A)}{-m K r_L} \quad (r_L < 0), \end{aligned} \end{aligned}$$

where $U_1$ arises from the effective stiffness condition ($a_4>0$) and $U_2$ from heave-pitch coupling ($\Delta _2>0$).

**Fig. 2:**
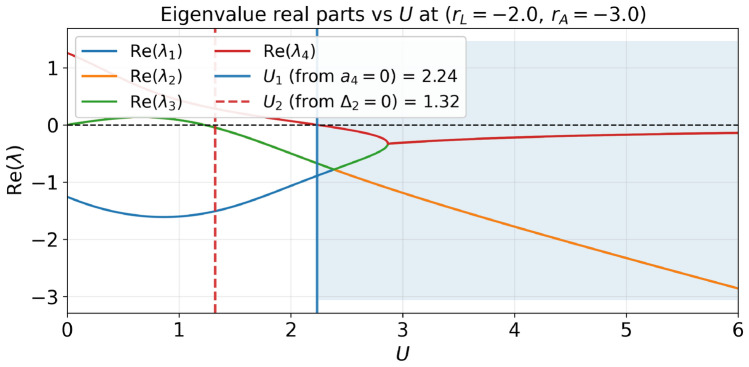
Real parts of the characteristic eigenvalues $\lambda _i(U)$ versus forward speed *U* for fixed geometry $r_L = -2.5$, $r_A = -3.0$. Green shading indicates speed ranges where all $\Re (\lambda _i) < 0$ (stable regime). The system is unstable at *U=0*, stabilizes beyond *U ≈ 1.2*, and undergoes oscillatory instability at *U ≈ 3.5* due to geometric destabilization.

To illustrate the stability transitions directly, Fig. 2 shows the real parts of all four eigenvalues as a function of forward speed *U* for a representative tail geometry ($r_L = -2.5$, $r_A = -3.0$). The system exhibits instability at *U=0* (one positive real eigenvalue), stabilizes beyond the lower threshold *U ≈ 1.2* where all $\Re (\lambda ) < 0$, and eventually loses stability again via an oscillatory mode (complex conjugate pair crossing zero) at *U ≈ 3.5*, consistent with violation of the geometric condition $B(r_L)<0$.

**Fig. 3:**
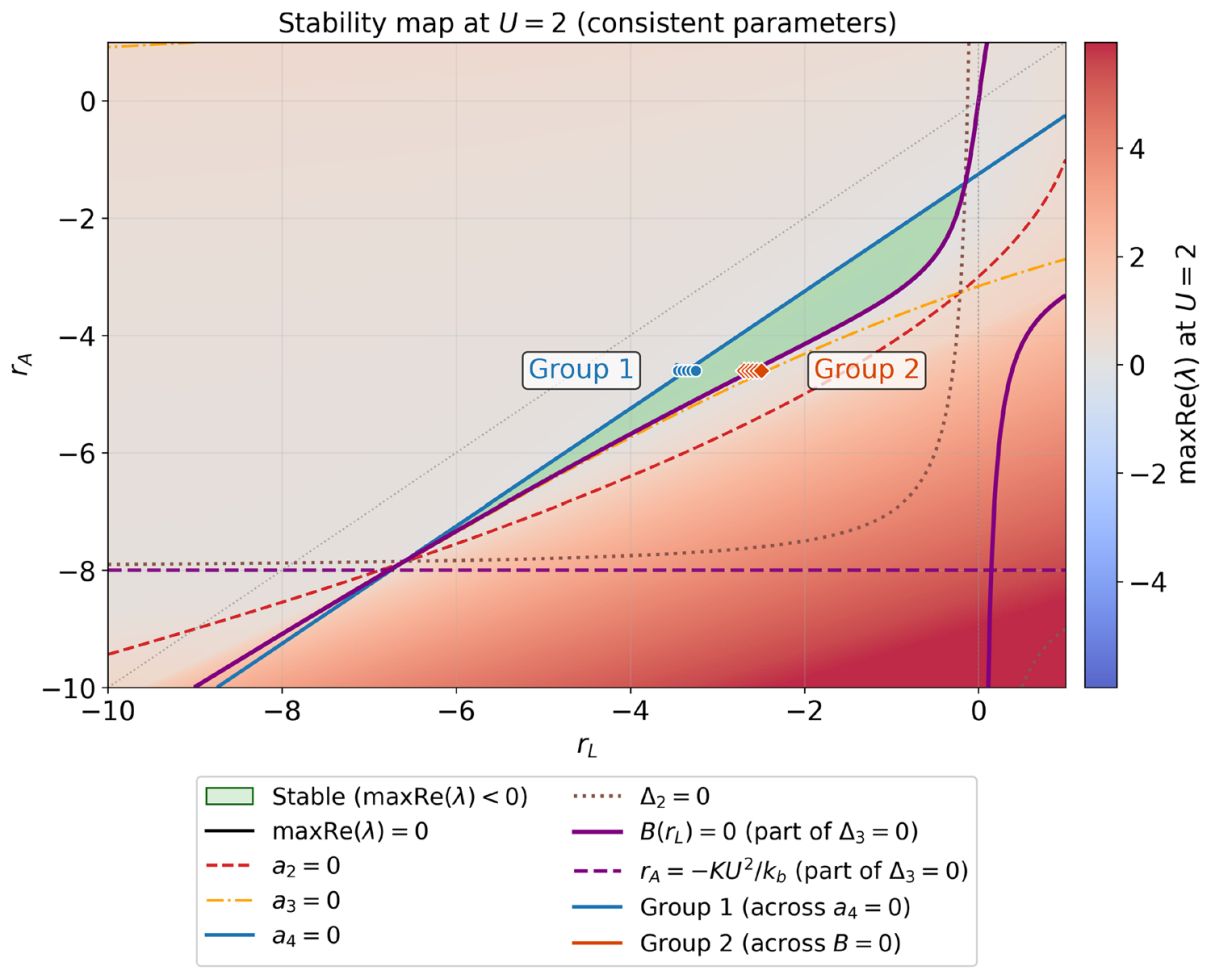
Comprehensive stability map in the $(r_L, r_A)$ plane at *U=2.0*, showing the stable region (green) and zero-level curves of all Routh–Hurwitz minors. The purple curves ($B(r_L)=0$ solid, $r_A=-KU^2/k_b$ dashed) and blue curve ($a_4=0$) represent decisive stability boundaries. Two transition groups are marked: Group 1 (blue arrow, aperiodic transition across $a_4=0$) and Group 2 (orange arrow, oscillatory transition across $B(r_L)=0$), whose dynamic responses are examined in Fig. 4.

Figure 3 generalizes this analysis across different geometries, showing the complete stability landscape in the $(r_L, r_A)$ plane at fixed speed *U=2.0* with all Routh–Hurwitz minor zero-curves. The stable region (green) requires $r_A< r_L < 0$ and satisfies all Routh–Hurwitz conditions. Two transition groups are highlighted: Group 1 (aperiodic transition across $a_4=0$) and Group 2 (oscillatory transition across $B(r_L)=0$), with arrows indicating the direction of parameter variation.

**Fig. 4:**
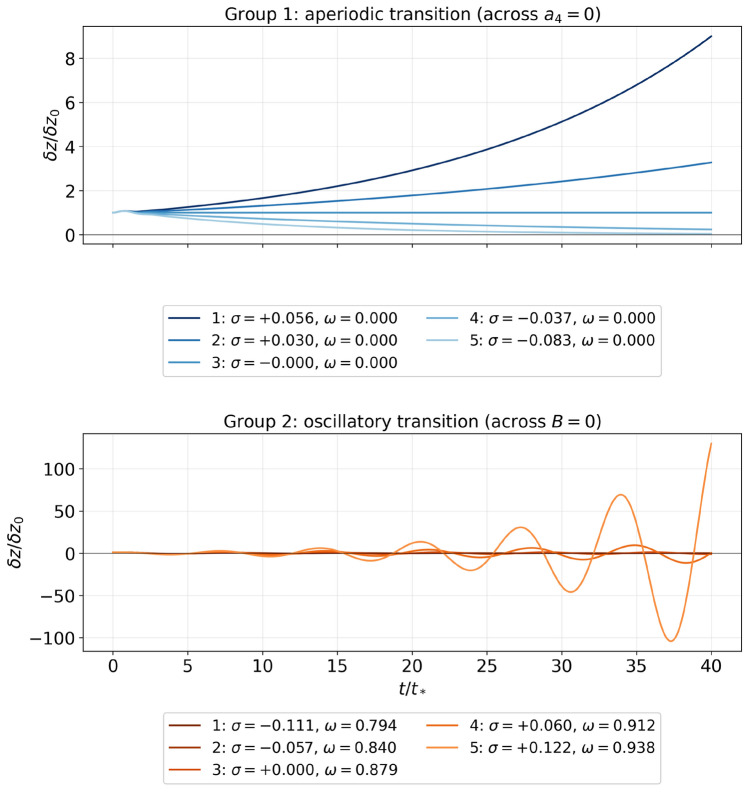
Time evolution of normalized depth perturbation $\delta z(t)/\delta z_0$ for the two transition groups marked in Fig. 3. **(Top)** Group 1: aperiodic transition across the $a_4=0$ boundary, showing progression from monotonic instability (points 1-2, *σ >0*, *ω =0*) to stable decay (points 4-5, *σ <0*). **(Bottom)** Group 2: oscillatory transition across the $B(r_L)=0$ boundary, showing progression from stable oscillatory decay (points 1-2, *σ <0*, *ω >0*) to oscillatory instability (points 4-5, *σ >0*, *ω >0*). The legend indicates growth/decay rates (*σ*) and frequencies (*ω*) for each trajectory.

To visualize the dynamic regimes near these boundaries, we simulate the full linearized system for each group. Figure 4 shows the time evolution of depth perturbation *δ z(t)* for both groups. In Group 1, we observe a transition from monotonic instability (trajectories 1–2) to stable decay (trajectories 4–5) as we cross the $a_4=0$ boundary. In Group 2, the transition is from stable decay (trajectories 1–2) to oscillatory instability (trajectories 4–5) as we cross the $B(r_L)=0$ boundary. The growth/decay rates (*σ*) and frequencies (*ω*) for each trajectory confirm the nature of the stability transitions.

**Fig. 5:**
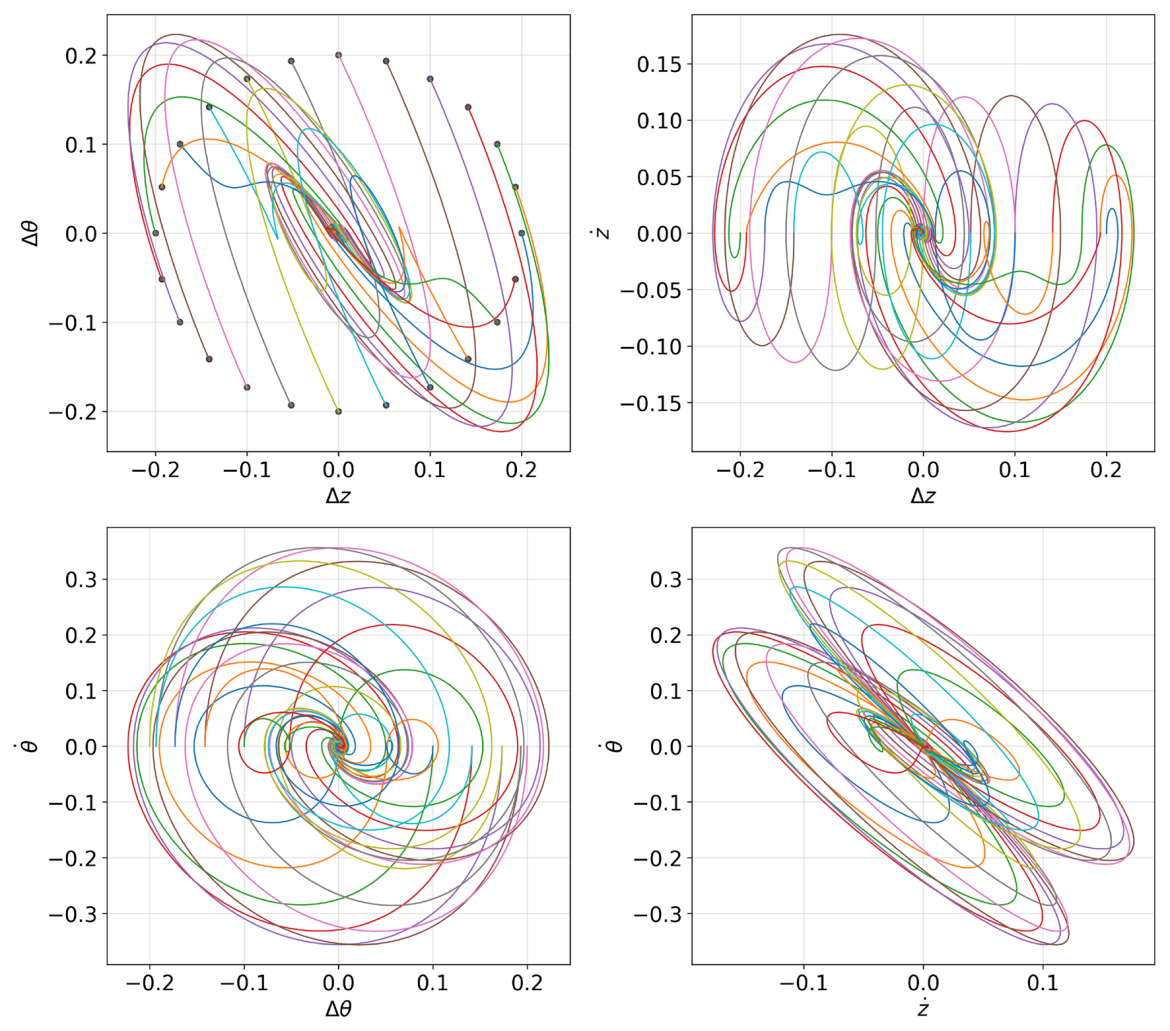
Phase portrait projections for stable point A in various state-space planes: (**a**) $(z,\dot{z})$ - vertical motion, (**b**) $(\theta ,\dot{\theta })$ - rotational motion, (**c**) *(z,θ )* - depth-orientation coupling, (**d**) $(\dot{z},\dot{\theta })$ - vertical and angular velocity coupling. All trajectories show spiral convergence to the origin, confirming asymptotic system stability.

For detailed analysis of stable dynamics at a representative point, we further investigate phase trajectories in the full four-dimensional state space. Figure 5 presents projections of phase portraits onto various two-dimensional planes: $(z,\dot{z})$, $(\theta ,\dot{\theta })$, *(z,θ )*, and $(\dot{z},\dot{\theta })$. All trajectories demonstrate characteristic spiral convergence to the origin, confirming asymptotic stability of the system in this parameter region. Particularly indicative is the behavior in the *(z,θ )* plane, where a weak oscillatory mode due to interaction between vertical motion and pitch is clearly visible.

These numerical experiments confirm the analytical stability boundaries and illustrate two fundamentally different mechanisms of stability loss described by our model.

**Classification of the equilibrium.** For the linear system $\dot{x}=A(U)x$, the origin is classified via the spectra of *A*(*U*) and the Routh–Hurwitz test.

In the *stable* region–defined by the two decisive inequalities $a_4>0$ and $\Delta _3>0$–all eigenvalues have negative real parts, so $\dim W^s=4$ and $\dim W^u=0$. These two inequalities are equivalent to the speed bound $U>\sqrt{k_\theta /[K(r_L-r_A)]}$ (requiring $r_L>r_A$) together with the mixed speed–geometry condition $(KU^2+k_b r_A)\,B(r_L)<0$ (here $B(r_L)$ is the geometric factor introduced above).

At *U=0* one eigenvalue is positive, one negative, and a conjugate pair is purely imaginary; hence $\dim W^s=1$, $\dim W^u=1$, and $\dim W^c=2$ (saddle–center). For $0<U<U_{\min }$ the number of unstable modes is 1 or 2 depending on parameters (as decided by the Routh–Hurwitz inequalities).

On the boundaries, different crossings occur: at $U=U_1$ (i.e., $a_4=0$) a simple zero eigenvalue appears, while along $\Delta _3=0$–either $r_A=-KU^2/k_b$ or $B(r_L)=0$–a conjugate imaginary pair crosses the axis, so $\dim W^c>0$.

By contrast, the conditions $a_2=0$ or $\Delta _2=0$ do not generically correspond to the observed spectral crossings in this model and are not active in the parameter ranges of interest.

## Analytical optimum

In the design of underwater vehicles, it is crucial to be able to analytically estimate the minimum allowable parameters and find optimal coefficient combinations.

We can analytically minimize $U_{\min }$ over all lever arms $(r_L,r_A)$ in the admissible tail geometry. Setting $r_A = -L$ (*L > 0*) and $r_L = -\alpha L$ (*0< α < 1*), the minimizer for fixed *α* satisfies $U_1 = U_2$. The remaining one-parameter problem has an interior optimum at

6 $$\begin{aligned} \alpha ^* = \frac{|r_L|}{|r_A|} = \sqrt{\frac{\kappa }{1 + \kappa }}, \qquad \kappa = \frac{k_b J}{m k_\theta }, \end{aligned}$$

approached from the stable side $B(r_L) \rightarrow 0^{-}$. The resulting *absolute* threshold speed, independent of $(r_L,r_A)$, is

7 $$\begin{aligned} U_{\min }^{2} = \frac{\sqrt{k_b k_\theta }}{K} \left( \sqrt{1 + \kappa } + \sqrt{\kappa }\right) , \qquad \kappa = \frac{k_b J}{m k_\theta }. \end{aligned}$$

## Simulation of nonlinear model

**Fig. 6:**
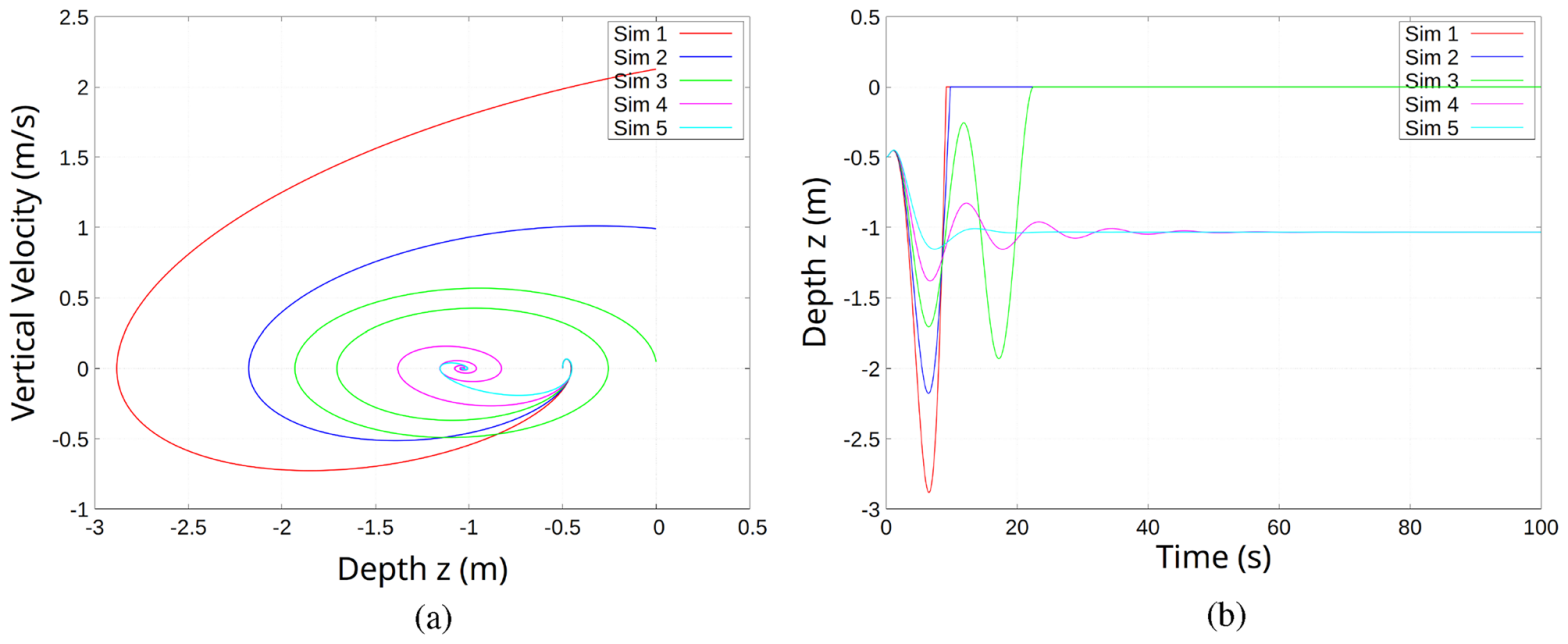
(**a**) Phase trajectory projections onto plane $(z,\dot{z})$ and (**b**) time dependence of cruising depth for different parameter values crossing the stability boundary at $r_A=-4.6$ m, Sim 1: $r_L=-2.45$ m; Sim 2: $r_L=-2.55$ m; Sim 3: $r_L=-2.65$ m; Sim 4: $r_L=-2.75$ m; Sim 5: $r_L=-2.85$ m.

Finally, we verify the stability analysis in direct simulation of a nonlinear model for arbitrary angles by changing $\theta \rightarrow \sin {\theta }$ in Eqs. (1–4). Crossing the stability boundary led to oscillatory instability and oscillations with increasing amplitude. For this particular parameter set we did not find a stable limit cycle typical for supercritical Andronov-Hopf bifurcation. For decreasing $r_L$ (curves Sim1-Sim3 in Fig. 6) the vehicle surfaces, reaching zero depth as illustrated in Fig. 6b.

**Fig. 7:**
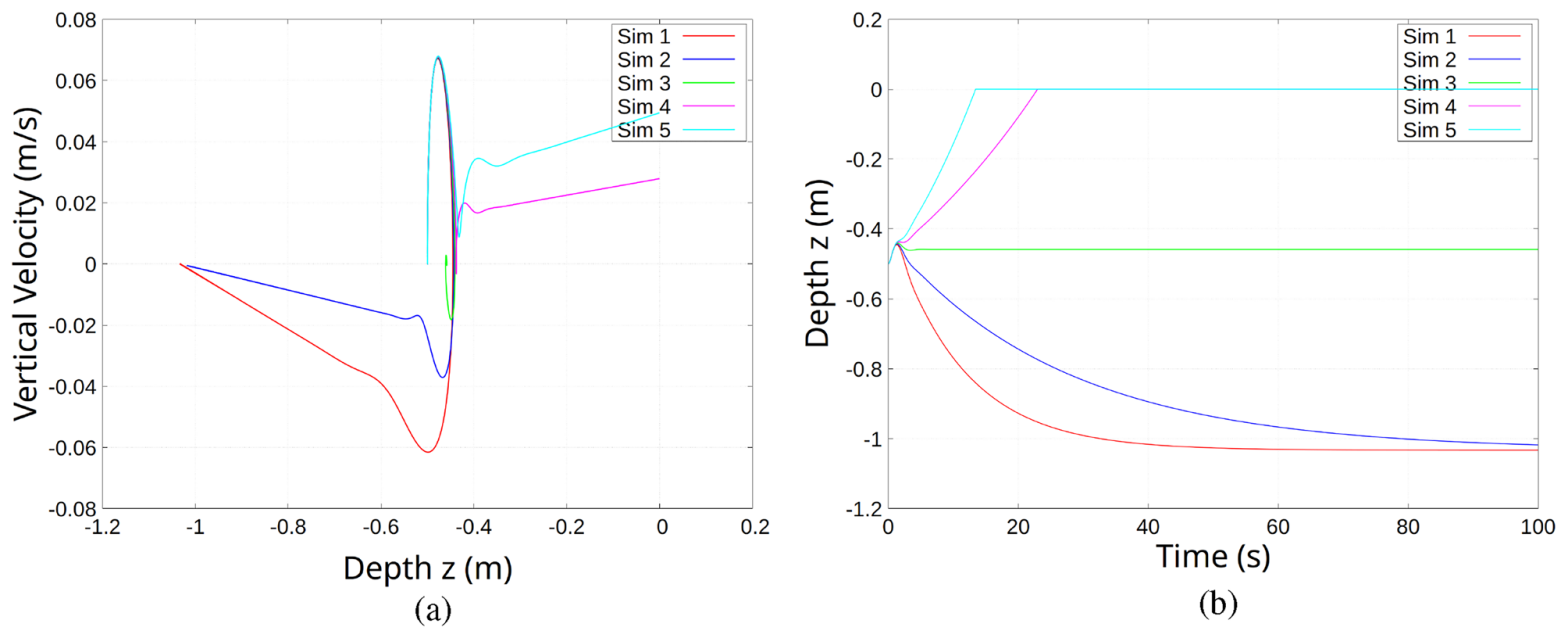
(**a**) Phase trajectory projections onto plane $(z,\dot{z})$ and (**b**) time dependence of cruising depth for different parameter values crossing the left stability boundary at $r_A=-4.6$ m, Sim 1: $r_L=-3.15$ m; Sim 2: $r_L=-3.25$ m; Sim 3: $r_L=-3.35$ m; Sim 4: $r_L=-3.45$ m; Sim 5: $r_L=-3.55$ m.

Figure 7 illustrates the vehicle dynamics near the left boundary of the stability region. In this case the values of the parameter $r_L$ were varied in the range from *-3.55* to *-3.15* m with a step of 0.1 m. Trajectories for $r_L = -3.55$ m (Sim5) and $r_L = -3.45$ m (Sim4), similarly to the right boundary, demonstrate unstable behavior—the vehicle is unable to counteract the disturbance and transitions into an ascent mode up to zero depth. Trajectories for $r_L = -3.25$ m (Sim2) and $r_L = -3.15$ m (Sim1) converge to an equilibrium point, confirming the vehicle’s ability to achieve dynamic stabilization for these values of the geometric parameter.

Thus, simulation of the nonlinear model confirmed the correctness of the stability boundaries. It was also demonstrated that the bifurcation scenario of stability loss is likely subcritical, as trajectories leave the vicinity of the fixed point when it becomes unstable.

## Discussion

This study distills the physics of depth stabilization with a *passive* swim bladder and forward motion into two decisive checks from the Routh–Hurwitz test. In practice, stability is governed by an effective stiffness condition (our $a_4>0$), which creates a lower onset speed, and a mixed speed-geometry condition (our $\Delta _3>0$), which ties the direction of motion to how lift and buoyancy are applied relative to the center of mass. Together, these two conditions reproduce the observed boundary on the full stability map (Fig. 3) without the need for extensive tuning parameters or active control effort.

The emerging physical picture is transparent. At low speed, the bladder’s compressibility softens the angular restoring effect and the system cannot maintain depth; as speed increases, flow-induced coupling between pitch and vertical motion provides the missing stiffness and enables stabilization. Geometry plays a crucial role: the $\Delta _3$ condition splits into a purely speed-related multiplier and a geometric factor that must remain negative ($B(r_L) < 0$) for stable operation. This imposes strict geometric constraints requiring both the buoyancy center and lift application point to be located tailward of the center of mass ($r_A< r_L < 0$).

Notably, these geometric requirements are more stringent than those found in biological systems like pike, where fins positioned toward the head can provide stabilization through active control mechanisms. In our model with fixed, body-aligned fins and purely passive dynamics, successful stabilization demands careful adherence to the tailward configuration rule. Direct transfer of biological morphologies without accounting for active control may therefore not yield stability, though incorporating active elements presents a promising direction for extending the present framework.

The proposed passive mechanism is not intended to replace active control entirely but rather to serve as an energy-efficient baseline. In practical applications, such as long-duration missions, the passive stabilization could maintain depth during steady cruising, while an auxiliary active controller would engage for maneuvers, trajectory tracking, or compensation of significant disturbances. This hybrid concept aligns with the growing interest in resource-aware and event-triggered control strategies^22^ that minimize communication and computational load. For instance, a lightweight adaptive controller, like the one presented by^23^, could be integrated to handle speed variations or unmodeled hydrodynamics beyond the passive stability region. Similarly, the design principles could be applied to biomimetic vehicles using CPG-based locomotion^13,19^, where the passive bladder would reduce the demand on the active depth control system.

For practical design, the guidelines are straightforward. First, separate the lift and bladder application points to satisfy the stiffness condition at the intended cruise speed (blue boundary in Fig. 3); this lowers the onset speed without continuous actuation. Second, position the bladder tailward and select the lift lever such that the operating point lies on the stabilizing side of the geometric curve determined by the $\Delta _3$ condition (purple curve).

**Limitations and outlook.** Our analysis is local (small angles, linearization about trim), assumes quasi-steady lift scaling with speed and a locally constant bladder stiffness, and neglects depth- and speed-dependent hydrodynamic variations. The identified speed threshold may be sensitive to unmodeled effects such as added mass, damping variations, and parameter uncertainties. Future work should include a sensitivity or robustness analysis to quantify these effects. Moreover, while internal numerical validation is provided, comparison with higher-fidelity simulation models or experimental data from a physical prototype, following the approach of Plamondon^19^, is necessary to fully establish engineering relevance. Extending the framework to weakly nonlinear effects near the boundaries, depth-varying bladder compliance, added-mass/viscous corrections, and in situ experiments on a physical prototype are natural next steps. Incorporating slowly time-varying cruise speed and three-dimensional couplings will further sharpen the engineering guidelines. Finally, exploring the integration of this passive module into more complex multi-vehicle systems or formations, where communication and coordination are critical^20,21^, presents an exciting avenue for future research.

## Conclusion

We have shown that a biomimetic vehicle with a *passive* swim bladder can stabilize depth purely through forward motion when geometry is chosen appropriately. The stability boundary collapses to two compact criteria–the onset set by an effective stiffness check and an oscillatory boundary set by a mixed speed–geometry check–which yield explicit, engineering-ready maps of admissible lever arms and operating speeds (Fig. 3). Direct numerical simulations of the linearized model corroborate these predictions and clarify the two dominant loss-of-stability scenarios: loss of stiffness at low speed and a geometry-controlled oscillatory crossing at higher speed. These results provide a concise, actionable basis for designing low-power, passively stable underwater vehicles and motivate experimental validation and refinement under realistic hydrodynamic conditions.

## Data Availability

All data available in repository with open access: https://github.com/JunFin/FishModel.
